# Glucose Amplifies Fatty Acid-Induced Endoplasmic Reticulum Stress in Pancreatic β-Cells *via* Activation of mTORC1

**DOI:** 10.1371/journal.pone.0004954

**Published:** 2009-03-23

**Authors:** Etti Bachar, Yafa Ariav, Mali Ketzinel-Gilad, Erol Cerasi, Nurit Kaiser, Gil Leibowitz

**Affiliations:** Endocrinology and Metabolism Service, Department of Medicine, Hadassah - Hebrew University Medical Center, Jerusalem, Israel; University of Bremen, Germany

## Abstract

**Background:**

Palmitate is a potent inducer of endoplasmic reticulum (ER) stress in β-cells. In type 2 diabetes, glucose amplifies fatty-acid toxicity for pancreatic β-cells, leading to β-cell dysfunction and death. Why glucose exacerbates β-cell lipotoxicity is largely unknown. Glucose stimulates mTORC1, an important nutrient sensor involved in the regulation of cellular stress. Our study tested the hypothesis that glucose augments lipotoxicity by stimulating mTORC1 leading to increased β-cell ER stress.

**Principal Findings:**

We found that glucose amplifies palmitate-induced ER stress by increasing IRE1α protein levels and activating the JNK pathway, leading to increased β-cell apoptosis. Moreover, glucose increased mTORC1 activity and its inhibition by rapamycin decreased β-cell apoptosis under conditions of glucolipotoxicity. Inhibition of mTORC1 by rapamycin did not affect proinsulin and total protein synthesis in β-cells incubated at high glucose with palmitate. However, it decreased IRE1α expression and signaling and inhibited JNK pathway activation. In TSC2-deficient mouse embryonic fibroblasts, in which mTORC1 is constitutively active, mTORC1 regulated the stimulation of JNK by ER stressors, but not in response to anisomycin, which activates JNK independent of ER stress. Finally, we found that JNK inhibition decreased β-cell apoptosis under conditions of glucolipotoxicity.

**Conclusions/Significance:**

Collectively, our findings suggest that mTORC1 mediates glucose amplification of lipotoxicity, acting through activation of ER stress and JNK. Thus, mTORC1 is an important transducer of ER stress in β-cell glucolipotoxicity. Moreover, in stressed β-cells mTORC1 inhibition decreases IRE1α protein expression and JNK activity without affecting ER protein load, suggesting that mTORC1 regulates the β-cell stress response to glucose and fatty acids by modulating the synthesis and activity of specific proteins involved in the execution of the ER stress response. This novel paradigm may have important implications for understanding β-cell failure in type 2 diabetes.

## Introduction

In type 2 diabetes mellitus (T2DM), elevated blood glucose and free-fatty acids (FFAs) induce β-cell dysfunction and apoptosis leading to exacerbation and progression of diabetes, a process called glucolipotoxicity [1]. High levels of saturated but not monounsaturated fatty acids were reported to increase β-cell apoptosis in rat and human islets [2], [3], [4], [5]. However, the toxic effect of FFAs on the pancreatic β-cells, originally termed lipotoxicity, gains pathological significance mainly under the *in vivo* hyperglycemic state [6], [7]. Thus, glucose seems to be an important amplifier of lipotoxicity. The mechanisms underlying this effect of glucose are not entirely clear.

There is ample evidence that palmitate induces β-cell dysfunction and apoptosis *via* activation of ER stress [8], [9], [10], [11], probably due to alteration of β-cell calcium fluxes and down-regulation of carboxypeptidase E [12], which perturbs the folding and maturation of secreted and membrane-bound proteins in the ER. This activates a complex signaling network called the unfolded protein response (UPR) aimed at adaptation and restoration of normal ER function, pursued by translation attenuation, degradation of misfolded proteins and increased protein folding capacity through augmented transcription of ER chaperones, such as BIP. When the UPR fails to restore adequate ER function it turns on signaling pathways leading to apoptosis [11], [13], [14].

The UPR involves three major signaling pathways initiated by three ER transmembrane sensor proteins: IRE1 (inositol requiring ER-to nucleus signal kinase 1), the pancreatic ER kinase PERK (double stranded RNA-activated protein kinase-like ER-associated kinase) and ATF6 (activating transcription factor 6) [15], [16], [17]. IRE1 activates the c-Jun N-terminal kinase (JNK) pathway; its sustained activation leads to apoptosis [18]. In addition, IRE1 cleaves the mRNA of the X-box binding protein-1 (Xbp-1) transcription factor. Spliced Xbp-1 (Xbp-1s) is an important regulator of ER folding capacity [19], [20]. Activation of PERK leads to phosphorylation of eukaryotic translation initiation factor 2 alpha (eIF2α) leading to attenuation of translation under ER stress conditions [21]. Activation of the PERK-eIF2α and ATF6 pathways may induce apoptosis through the transcriptional activation of the CCAAT/enhancer binding protein homologous protein (CHOP) gene [22]. Collectively, inducers of ER stress cause apoptosis through activation of JNK and CHOP.

The mammalian target of rapamycin (mTOR), a conserved serine/threonine kinase, functions as an important nutrient sensor; its downstream effectors regulate protein translation and cell growth, proliferation and survival [23], [24]. mTOR exists in two distinct complexes: a rapamycin-sensitive complex called mammalian target of rapamycin complex 1 (mTORC1), which includes the regulatory associated protein of mTOR (raptor) and mLST8/GbL, and in a rapamycin-resistant complex, mammalian target of rapamycin complex 2 (mTORC2) with rapamycin insensitive companion of mTOR (rictor), mLST8/GbL and Sin1 [25]. Exposure of β-cells to hyperglycemia *in vivo* and *in vitro* results in marked activation of mTORC1, which induces phosphorylation of ribosomal S6 kinases (S6K1 and S6K2) and the translation inhibitor factor, eukaryotic initiation factor 4E binding protein 1 (4EBP1) [26]. S6K phosphorylates the 40S ribosomal protein S6, the protein synthesis initiation factor 4B (eIF4B) and the elongation factor 2 kinase (eEF2K) [27]. 4EBP1 phosphorylation releases eIF4E from 4EBP1, allowing cap-dependent translation initiation [26], [28]. The activation of S6K1/2 and the release of eIF4 by mTORC1 stimulate ribosomal biogenesis and mRNA translation, hence augment protein biosynthesis.

It appears from the above that mTORC1 stimulation of protein synthesis might counteract the adaptive attenuation of protein synthesis under conditions of ER stress. Herein we tested the hypothesis that glucose augments lipotoxicity by stimulating mTORC1, thus leading to increased FFA-induced ER stress, with augmentation of β-cell apoptosis as a consequence.

## Results

### The role of mTORC1 in glucose- and palmitate-induced β-cell apoptosis

**Figure 1:**
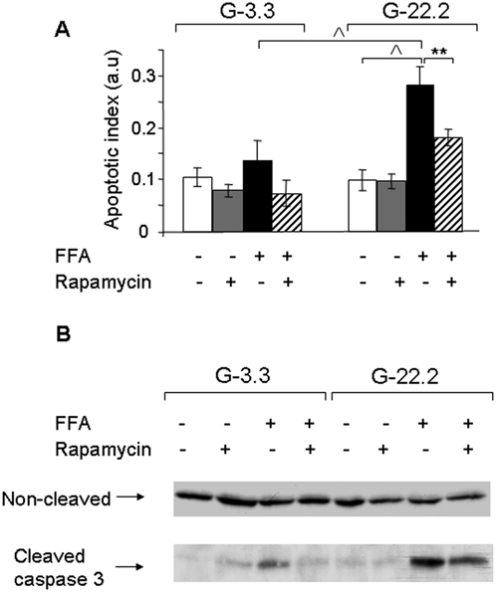
Effect of mTORC1 inhibition by rapamycin on glucose and palmitate-induced β-cell apoptosis. INS-1E cells were incubated at 3.3 and 22.2 mmol/l glucose with 0.5% BSA with and without 0.5 mmol/l palmitate and 50 nmol/l rapamycin for 16 h. Apoptosis was assessed using the Cell Death ELISA^PLUS^ assay (Roche Diagnostics) (A) and by Western blot for cleaved caspase 3 (B). Results are expressed as means±SE of 4 individual experiments, each performed in triplicates (A). A representative gel of 3 individual experiments showing the expression of cleaved and uncleaved caspase 3 is presented (B). ** p<0.01, ^∧^ p<0.001 for the difference between the indicated groups.

INS-1E β-cells were treated with 0.5 mmol/l palmitate at 3.3 and 22.2 mmol/l glucose for 16 h. Apoptosis was quantified using an ELISA assay for cytosolic oligonucleosome content indicative of apoptosis-induced DNA degradation (Figure 1A). At 3.3 mmol/l glucose, palmitate had a small, non-significant effect on β-cell apoptosis; the tendency for apoptosis was 1.4-fold higher than in untreated cells incubated at 0.5% BSA (w/v). Incubation of INS-1E cells at 22.2 mmol/l sensitized the β-cells to the toxic effect of palmitate: the apoptotic rate of INS-1E β-cells treated with palmitate was now 2.1- and 2.9-fold higher than that of palmitate-treated cells at 3.3 mmol/l glucose and untreated controls at 22.2 mmol/l glucose, respectively.

To study the role of mTORC1 in glucose-induced palmitate toxicity we used the mTORC1 inhibitor rapamycin. Rapamycin prevented β-cell apoptosis in response to palmitate both at low and high glucose (Figure 1A). Western blot analysis showed that 22.2 mmol/l glucose did not affect caspase 3 cleavage. Palmitate modestly increased cleaved caspase 3 at 3.3 mmol/l glucose; whereas, it markedly augmented caspase 3 activation at 22.2 mmol/l glucose (Figure 1B). Consistent with the anti-apoptotic effect of rapamycin in β-cells exposed to glucolipotoxicity conditions, we found that rapamycin attenuated caspase 3 cleavage in INS-1E cells incubated at 22.2 mmol/l glucose with palmitate (Figure 1B). Rapamycin did not decrease the rate of β-cell proliferation (Figure S1). Taken together, our data suggest that mTORC1 is involved in palmitate-induced β-cell apoptosis and its amplification by glucose.

### Glucose and palmitate effects on mTORC1 signaling

**Figure 2:**
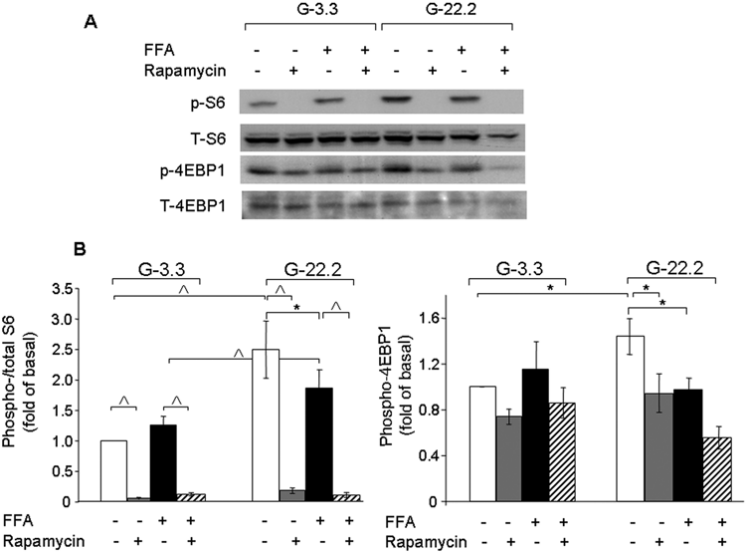
Effects of glucose, palmitate and rapamycin on mTORC1 signaling in β-cells. INS-1E cells were incubated overnight in RPMI medium containing 3.3 mmol/l glucose and 0.5% BSA without serum and then at 3.3 and 22.2 mmol/l glucose with and without 0.5 mmol palmitate and 50 nmol/l rapamycin for 4 h. S6(Ser235/236) and 4EBP1 phosphorylation were analyzed by Western blot. (A) A representative gel showing total and phosphorylated S6 and 4EBP1 is presented. (B) Quantification of S6 and 4EBP1 phosphorylation. Results are expressed as means±SE of 6 individual experiments. * p<0.05, ^∧^ p<0.001 for the difference between the indicated groups.

Since mTORC1 may function as an important modulator of glucolipotoxicity-induced β-cell apoptosis, we studied the effects of high glucose and palmitate on its signaling in β-cells. Glucose increased S6 and 4EBP1 phosphorylation (Figure 2), showing as expected, that glucose activates mTORC1 signaling. Palmitate partially reduced mTORC1 activation by high glucose, as seen by reduced S6 and 4EBP1 phosphorylation levels. However, mTORC1 activity was higher in β-cells treated with palmitate at 22.2 mmol/l than at 3.3 mmol/l glucose. Rapamycin completely abolished S6 phosphorylation and partially decreased 4EBP1 phosphorylation under all conditions, indicating that rapamycin efficiently inhibited mTORC1 in INS-1E cells exposed to high glucose and palmitate.

### The role of mTORC1 in glucose- and palmitate-induced ER stress

**Figure 3:**
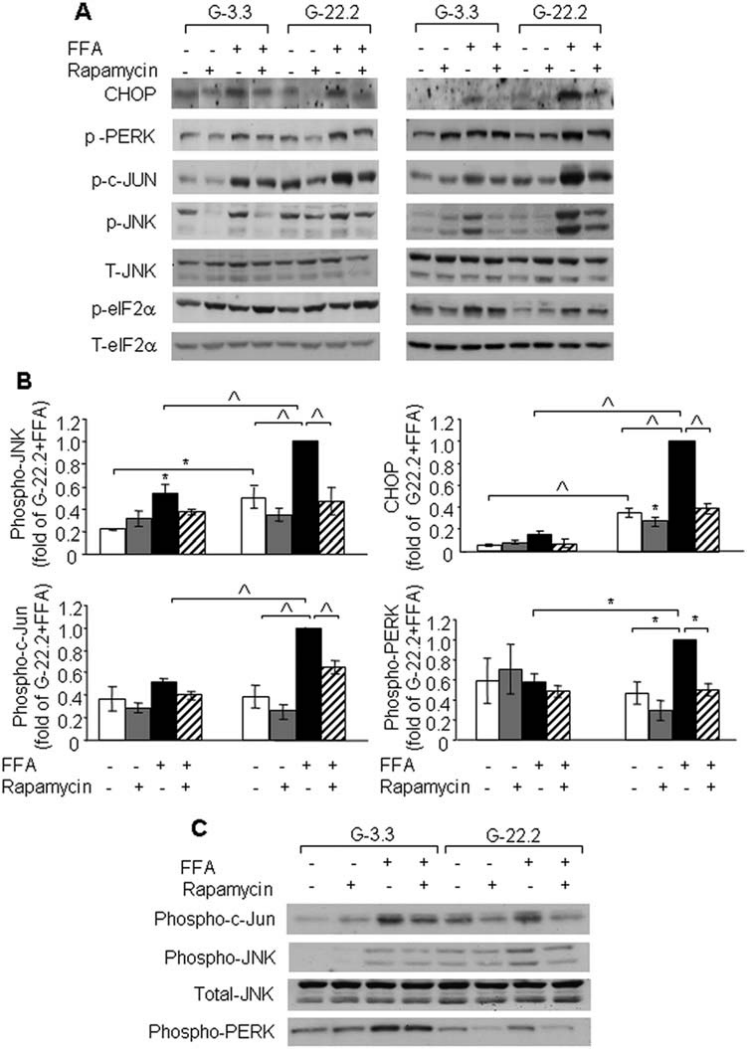
Effect of mTORC1 inhibition by rapamycin on glucose and palmitate-induced ER stress in β-cells and islets. INS-1E cells were incubated at 3.3 and 22.2 mmol/l glucose with and without 0.5 mmol/l palmitate and 50 nmol/l rapamycin for 4 and 16 h; *P. obesus* islets were similarly treated for 24 h. Control incubations contained 0.5% BSA. ER stress was assessed by Western blot analysis for different ER stress markers. (A) A representative gel showing CHOP, phospho-PERK, phospho-c-Jun, phospho- and total JNK, phospho- and total eIF2α in INS-1E cells is presented. (B) Quantification of c-Jun, JNK, and PERK phosphorylation and CHOP expression at 16 h. Results are expressed as means±SE of 4 individual experiments. (C) A representative gel of 3 individual experiments showing phospho-c-Jun, phospho-PERK and phospho- and total JNK in *P. obesus* islets. * p<0.05, ^∧^ p<0.001 for the difference between the indicated groups or between the indicated groups and untreated controls at the same glucose concentration.

Palmitate induces β-cell apoptosis through activation of ER stress [8], [9], [10], [11]. We studied whether glucose increased palmitate toxicity through amplification of the ER stress response. Following a 4-h incubation, palmitate stimulated different ER stress markers including CHOP, phospho-PERK, c-Jun, JNK and eIF2α (Figure 3A). c-Jun and JNK phosphorylation was higher at 22.2 mmol/l than at 3.3 mmol/l glucose. Importantly, the phosphorylation of these kinases was higher in palmitate-treated INS-1E cells at 22.2 mmol/l compared to 3.3 mmol/l glucose. However, the expression of the other ER stress markers was similar or only modestly increased in palmitate-treated INS-1E cells at 22.2 *vs* 3.3 mmol/l glucose (Figure 3A). Thus, glucose amplifies the early activation of the JNK pathway by palmitate with a small effect on the activation of other ER stress parameters. At 16 h, high glucose markedly increased palmitate activation of CHOP, PERK, c-Jun and JNK. The induction of these stress markers was decreased by rapamycin (Figure 3A&B).

*P. obesus* is an animal model of type 2 diabetes characterized by increased susceptibility to glucolipotoxicity-induced β-cell apoptosis [29]. Incubation of *P. obesus* islets at 22.2 mmol/l glucose increased palmitate-induced JNK phosphorylation compared to islets at 3.3 mmol/l glucose (Figure 3C). However, contrasting the amplifying effect of glucose on palmitate-induced PERK phosphorylation in INS-1E cells, in *P. obesus* islets glucose decreased PERK phoshorylation (Figure 3C). Thus, PERK phosphorylation is differentially regulated by glucose in the β-cell line and in *P. obesus* islets. Similar to INS-1E cells, rapamycin decreased the phosphorylation of JNK, c-Jun and PERK in islets under conditions of glucolipotoxicity.

In summary, rapamycin decreased the activation of ER stress markers by palmitate and attenuated the enhancement of ER stress and apoptosis by glucose.

The early activation of the JNK/c-Jun pathway by glucose in INS-1E cells and the finding that glucose increases palmitate-induced JNK phosphorylation also in islets led us to study further the role of JNK in β-cell ER stress and apoptosis under conditions of glucolipotoxicity.

### Regulation of the IRE1α-JNK pathway under conditions of glucolipotoxicity

**Figure 4:**
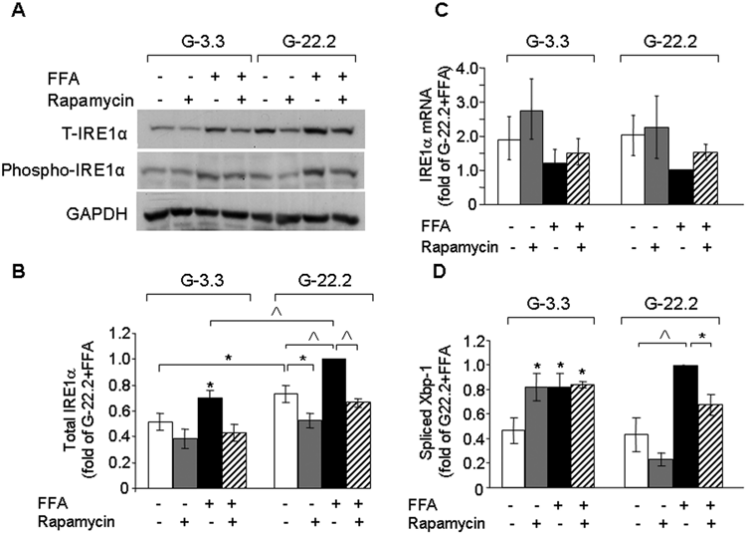
Effects of glucose, palmitate and rapamycin on IRE1α expression and activity. INS-1E cells were incubated at 3.3 and 22.2 mmol/l glucose with and without 0.5 mmol/l palmitate and 50 nmol/l rapamycin for 16 h. (A) A representative gel showing total and phospho-IRE1α. (B, C) Quantification of IRE1α protein and mRNA levels, respectively. (D) Spliced Xbp-1 levels assessed by quantitative real-time PCR. Results are expressed as means±SE of 4–5 individual experiments. * p<0.05, ^∧^ p<0.001 for the difference between the indicated groups or between the indicated groups and untreated controls at the same glucose concentration.

JNK is a direct target of IRE1α; therefore, we studied the effects of glucose, palmitate and rapamycin on IRE1α expression and function. IRE1α protein level was higher in INS-1E cells incubated at 22.2 mmol/l than at 3.3 mmol/l glucose. Palmitate increased IRE1α protein level at both glucose concentrations, whereas rapamycin decreased its expression (Figure 4A–B). The expression of other peptides involved in the regulation of ER stress, such as JNK and eIF2α was similar under all conditions (Figure 3A), indicating that glucose and palmitate stimulation of protein expression is specific to IRE1α. The time-course of IRE1α protein levels in β-cells treated with high glucose and palmitate showed that high glucose increased IRE1α expression in β-cells treated with or without palmitate for 8–16 h (Figure 4A–B and Figure S2A). This was paralleled by changes in IRE1α and JNK/c-Jun phosphorylation (Figure 4A and Figure 3). Next, we studied the effects of nutrients and rapamycin on IRE1α gene expression (Figure 4C and Figure S2B). IRE1α mRNA levels were similar under all treatment conditions, indicating that nutrients and mTORC1 regulate IRE1α protein expression at the post-tanscriptional level.

IRE1α functions as an endoribonuclease, which initiates the splicing of the mRNA encoding the key transcription factor XBP-1. Spliced Xbp-1 was not increased in palmitate-treated INS-1E cells at 22.2 mmol/l compared to similarly treated cells at 3.3 mmol/l glucose. Rapamycin decreased Xbp1 splicing in β-cells treated with palmitate at 22.2 mmol/l glucose (Figure 4D).

### mTORC1 regulation of proinsulin and global protein synthesis under conditions of glucolipotoxicity

**Figure 5:**
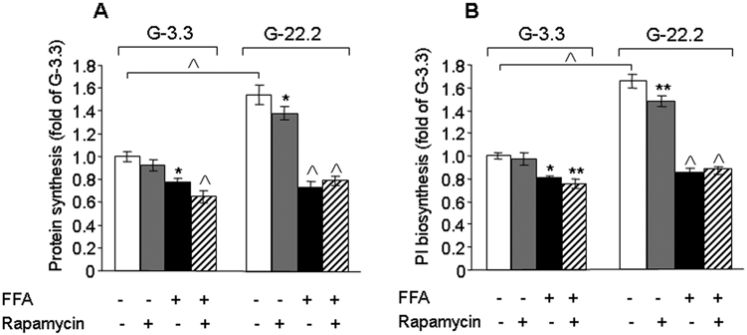
Effects of glucose, palmitate and rapamycin on total protein and proinsulin biosynthesis. INS-1E cells were incubated for 16 h at 3.3 and 22.2 mmol/l glucose with 0.5% BSA with and without 0.5 mmol/l palmitate and 50 nmol/l rapamycin. The last 2 h of the incubations were performed in KRBH-BSA buffer containing similar treatments and 10 µCi L-[2, 3, 4, 5-^3^H]leucine. After a 2-h incubation at 37°C, leucine incorporation was terminated by ice-cold wash-out in glucose-free KRBH-BSA buffer. Total protein synthesis (A) was determined by trichloroacetic acid precipitation. Proinsulin (PI) biosynthesis (B) was determined by immunoprecipitation with anti-insulin serum. Results are expressed as means±SE of 3 individual experiments, each performed in triplicates. * p<0.05, ** p<0.01, ^∧^ p<0.001 for the difference between the indicated groups or between the indicated groups and untreated controls at the same glucose concentration.

**Figure 6:**
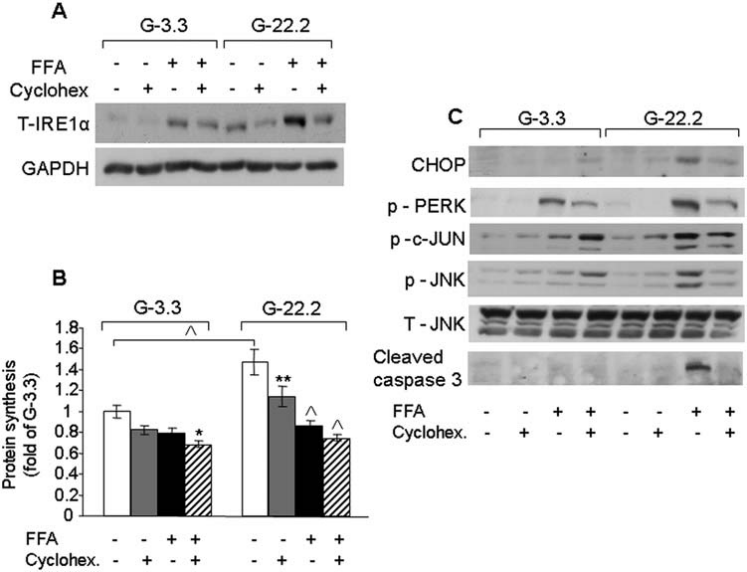
Effects of cycloheximide on IRE1α protein levels, total protein synthesis and different markers of ER stress in INS-1E cells treated with palmitate. INS-1E cells were incubated at 3.3 and 22.2 mmol/l glucose with and without 0.5 mmol/l palmitate and 20 nmol/l cycloheximide for 16 h. (A) IRE1α expression was analyzed by Western blot. A representative gel of 4 individual experiments is shown. (B) Total protein synthesis was determined as described in Figure 5. Results are expressed as means±SE of 3 individual experiments, each performed in triplicates. (C) Western blot analysis for different ER stress and apoptosis markers. A representative gel of 4 individual experiments showing CHOP, phospho-PERK, phospho-c-Jun, phospho- and total JNK and cleaved caspase 3 is presented. * p<0.05, ** p<0.01, ^∧^ p<0.001 for the difference between the indicated groups or between the indicated groups and untreated controls at the same glucose concentration.

Activation of mTORC1 augments protein synthesis, which may increase the ER client protein load, leading to exacerbation of ER stress. In β-cells, proinsulin is the most abundant protein delivered to the ER. Therefore, we studied the effects of glucose, palmitate and rapamycin on proinsulin and global protein synthesis in β-cells (Figure 5–6). Culture of INS-1E cells at 22.2 mmol/l glucose induced 1.5- and 1.7-fold increase in total protein and proinsulin biosynthesis, respectively. Strikingly, palmitate completely abolished the glucose stimulation of proinsulin and total protein biosynthesis, indicating that in β-cells the induction of ER stress by palmitate shuts off protein synthesis. Rapamycin decreased glucose-stimulated proinsulin and total protein biosynthesis only by 10% without affecting protein synthesis in palmitate-treated INS-1E cells.

### Effects of IRE1α inhibition by cycloheximide on ER stress

It is well established that rapamycin is an inhibitor of mRNA translation. To further study the effects of translation inhibition on IRE1α synthesis in stressed β-cells, INS-1E β-cells were incubated under glucolipotoxic conditions with and without a low concentration (0.2 mmol/l) of the translational inhibitor cycloheximide. Cycloheximide prevented glucose and fatty acid augmentation of IRE1α expression without affecting global protein synthesis in palmitate treated cells, mimicking the effects of rapamycin (Figure 6A–B).

We then studied whether translational inhibition of IRE1α by cycloheximide may alleviate the ER stress response in β-cells exposed to glucolipotoxic conditions. Cycloheximide decreased JNK, c-Jun and PERK phosphorylation, CHOP expression and cleaved caspase 3 levels in palmitate-treated β-cells at high glucose (Figure 6C), indicating that inhibition of translation alleviated the stress exerted on the ß-cells through inhibition of IRE1α expression, rather than reduction of ER protein load.

### mTORC1 regulation of JNK/c-Jun phosphorylation

Glucose robustly increased the stimulation of JNK/c-Jun phosphorylation by palmitate. This effect was rapid and preceded the glucose amplification of other ER stress markers (Figure 3). However, activation of the JNK pathway is not exclusive to ER stress. To confirm that mTORC1 regulates JNK/c-Jun activation in response to ER stress, we studied the effect of thapsigargin, which is an ER stressor, and that of anisomycin, which activates JNK in an ER stress-independent manner. This was studied in TSC2-deficient mouse embryonic fibroblasts (MEFs) and in wild-type TSC2 expressing cells. The TSC1-2 complex, through its Rheb-GTP activity, is a critical negative regulator of mTORC1 [30], [31]; therefore the mTORC1 pathway is constitutively active in TSC2-deficient cells.

**Figure 7:**
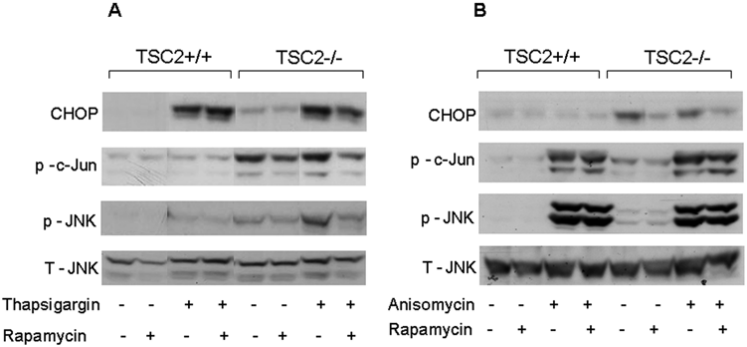
Effects of mTORC1 on ER stress-dependent and -independent JNK activation. TSC2-deficient and wild-type mouse embryonic fibroblasts were treated with 300 nmol/l thapsigargin for 24 h (A) or with 200 nmol/l anisomycin for 30 min (B) with and without 50 nmol/l rapamycin, as described under Materials and Methods. JNK and c-Jun phosphorylation were analyzed by Western blot. Representative gels of 3 individual experiments showing CHOP, phoshpo-c-Jun, total and phospho-JNK are presented.

Figure 7A), indicating activation of the ER stress response. Basal and thapsigargin-induced JNK and c-Jun phosphorylation was higher in TSC2−/− cells than in wild-type cells. Moreover, rapamycin decreased CHOP, JNK and c-Jun phosphorylation in the TSC2−/− MEFs (Figure 7A). Collectively, these findings indicate that dysregulated mTORC1 activity sensitizes cells to JNK activation by ER stress.

Anisomycin did not increase CHOP expression (Figure 7B), showing that it did not induce ER stress. As expected, anisomycin markedly increased JNK and c-Jun phosphorylation in both TSC2−/− and wild-type cells; however, rapamycin did not affect this activation, while reducing as expected CHOP expression. Taken together, these findings indicate that mTORC1 specifically regulates the JNK response to ER stress.

### The role of JNK in β-cell apoptosis under glucolipotoxic conditions

**Figure 8:**
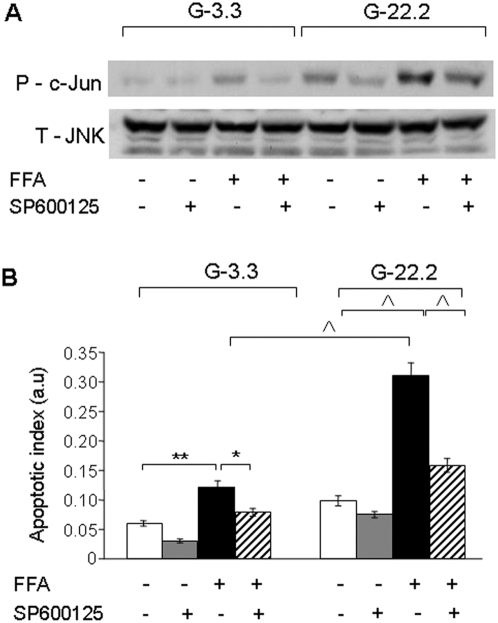
Effect of JNK inhibition on glucose and palmitate-induced β-cell apoptosis. INS-1E cells were incubated for 16 h at 3.3 and 22.2 mmol/l glucose with 0.5% BSA with and without 0.5 mmol/l palmitate and 20 nmol/l of the JNK inhibitor SP600125. (A) Inhibition of JNK activation was studied by Western blot for phospho-c-Jun normalized to total JNK. A representative gel of 3 separate experiments is shown. The effects of JNK inhibition on apoptosis was analyzed using the apoptosis ELISA assay (B). Quantification of 3 individual experiments, each performed in triplicates is shown. * p<0.05, ** p<0.01, ^∧^ p<0.001 for the difference between the indicated groups.

We studied the role of JNK in β-cell apoptosis in response to high glucose and palmitate. INS-1E cells were incubated at 3.3 and 22.2 mmol/l glucose with and without 0.5 mmol/l palmitate and 20 nmol/l of the JNK inhibitor SP600125. SP600125 completely inhibited the induction of c-Jun phosphorylation by palmitate at 3.3 mmol/l glucose. In addition, it partially inhibited c-Jun phosphorylation in response to 22.2 mmol/l glucose with and without palmitate (Figure 8A).

SP600125 decreased β-cell apoptosis of INS-1E cells under all conditions, with a dramatic 60% reduction of apoptosis in cells treated with palmitate at 22.2 mmol/l glucose (Figure 8B). These results clearly demonstrate that JNK inhibition can protect β-cells from glucolipotoxicity-induced apoptosis. SP600125 did not affect PERK phosphorylation and CHOP expression (Figure S3), indicating that its protective effect was through inhibition of JNK without affecting other ER stress pathways.

## Discussion

The main findings of this study are that: 1. glucose amplifies fatty acid-induced ER stress and apoptosis in β-cells; and 2. mTORC1 regulates the ER stress response under conditions of glucolipotoxicity through modulation of the IRE1α-JNK pathway without affecting global protein or proinsulin biosynthesis.

Herein, we show for the first time that glucose is a potent amplifier of the ER stress response to fatty acid. A previous study by Chunha et al reported that glucose does not amplify fatty acid-induced ER stress, contrasting our data [32]. The discrepancy between our data and those of Chunha et al may stem from the fact that in the previous report the expression of ER stress markers was analyzed mainly by qPCR, whereas we analyzed the protein levels and the phophorylation state of different mediators of the ER stress response. We believe that our data reflect ER stress activation more accurately, as the ER stress response is mediated through phosphorylation and activity of peptides. In this regard, it is of interest that the previous authors found that high glucose sensitized INS-1E cells to the apoptotic effect of a pharmacological inducer of ER stress [32], which may support our conclusion that glucose is an amplifier of ER stress.

Our findings emphasize the central role of glucose activation of the IRE1α-JNK pathway in glucolipotoxicity. This is based on detailed analysis of IRE1α expression and phosphorylation, JNK and c-Jun phosphorylation and Xbp-1 splicing. In our hands, the level of spliced Xbp-1 was the least sensitive marker for IRE1α kinase activity and did not completely overlap with the other indicators of IRE1α activation. Consistent with our findings, Lipson et al found that prolonged exposure of INS-1 cells to high glucose markedly increased IRE1α phosphorylation without affecting the level of spliced Xbp-1 [33]. Therefore, Xbp-1 should not be used as the sole parameter for assessing IRE-1α pathway activation.

High glucose levels markedly increase the demand on the β-cell ER due to increased synthesis of proteins, mostly proinsulin [34]. In rat islets, acute stimulation by glucose modestly increased several ER stress markers including spliced Xbp1, ER chaperones, ATF3, CHOP and GADD34 [35]. In addition, both short-term and prolonged exposure to a high glucose concentration were shown to induce IRE1 activation in mouse islets [33], [36]. However, the overall induction of ER stress by glucose is lower compared to that induced by palmitate, mainly under glucolipotoxic conditions.

Others and we have shown that high glucose activates mTORC1 in β-cells and islets (Figure 2) [37], [38]. Importantly, we found that inhibition of mTORC1 by rapamycin markedly decreased the ER stress response and apoptosis under conditions of glucolipotoxicity, indicating that mTORC1 is involved in the regulation of nutrient-induced ER stress in β-cells.

Our results are consistent with those of Ozcan *et al* who found that loss of tuberous sclerosis complex (TSC) suppressors in tumor cells, leading to dysregulated mTOR activity, triggers the UPR and increases apoptosis [39]. Our data extend these observations and suggest that mTOR regulates the ER stress response to nutrient overload in pathological metabolic states such as type 2 diabetes.

How does mTORC1 regulate the glucolipotoxic ER stress in β-cells? We tested the hypothesis that nutrient stimulation of mTORC1 amplifies the ER stress response by stimulating global protein, or specifically proinsulin biosynthesis. Palmitate is a potent suppressor of proinsulin and general protein synthesis in β-cells (Figure 5). This is achieved by IRE1-dependent degradation of ER-targeted mRNAs, such as proinsulin [33], [36]. In addition, PERK phosphorylates eIF2α, thereby inhibiting 80S ribosome assembly and consequently protein biosynthesis [21]. Global attenuation of protein biosynthesis through eIF2α paradoxically increases the expression of some distinct proteins including the transcription factor ATF4 [40]. It was recently shown that ATF4 induces 4EBP1, which inhibits protein synthesis and contributes to β-cell survival during late stages of the UPR [41]. Thus, induction of ER stress by palmitate turns on multiple pathways aimed to attenuate protein synthesis in β-cells exposed to glucolipotoxicity.

A recent quantitative assessment of the UPR showed that in mammalian professional secretory cells, such as pancreatic β-cells, translation attenuation is a critical adaptive mechanism to ER stress, which is less important in non-secretory cells and in lower eukaryotes [42]. In β-cells this adaptation is essential for survival under conditions of ER stress. Translation attenuation reduces the accumulation of unfolded proteins in the ER and the burden of chaperones that are long-lived proteins, which may cause cell toxicity during the recovery from stress [43].

Our study shows that in β-cells exposed to glucolipotoxic stress, the translation attenuation mechanism is highly efficient; the rate of protein biosynthesis was decreased to basal levels. Notably, rapamycin did not affect total protein or proinsulin biosynthesis under these conditions, indicating that in β-cells mTORC1 inhibition does not affect the translation attenuation induced by FFAs. Similarly, a low concentration of cycloheximide, mimicking the effects of rapamycin on protein synthesis at high glucose, efficiently abolished the ER stress response to glucose and palmitate without affecting global protein synthesis.

If this is the case, how does rapamycin reduce glucolipoxicity-induced ER stress in β-cells?

According to our results, it seems that rapamycin may attenuate the stress response to glucolipotoxicity by modulating the activity of specific pathways involved in the regulation of ER stress, notably the IRE1α-JNK pathway.

The JNK pathway is a critical mediator of fatty acid-induced ER stress, probably through activation of Foxo1 [44]. Glucose robustly increased JNK and c-Jun phosphorylation and amplified their stimulation by palmitate in an mTORC1-dependent manner. Moreover, JNK inhibition reduced β-cell apoptosis underscoring the important role of this pathway in mediating glucolipotoxicity-induced β-cell apoptosis.

It is important in this context, that IRE1α protein levels are increased by glucose and palmitate, an effect that could be prevented by rapamycin, indicating that mTORC1 is involved in the regulation of IRE1α biosynthesis. Glucose stimulation of c-Jun occurred early (at 4 h) and preceded the changes in IRE1α protein expression. Therefore, at early time points, glucose, palmitate and rapamycin may modulate JNK/c-Jun activation independent of their effects on IRE1α expression. However, at the late stage of the UPR (at 16 h) the changes in IRE1α protein levels are likely to be involved in the modulation of the IRE1α-JNK pathway activation.

We cannot exclude that rapamycin induced a small decrease of proinsulin or global protein synthesis, which was overlooked by the assay used in this study. However, the impact of such small inhibition of ER client protein load is most probably of a minor significance compared to the robust effects on the IRE1α-JNK pathway.

In summary, our data contradict the assumption that mTORC1 augments ER stress by increasing cell metabolism and protein synthesis and suggests instead that it regulates the β-cell stress response to glucose and fatty acids by modulating the synthesis and activity of specific proteins involved in the execution of the ER stress response, such as IRE1α.

### Implications for the pathogenesis and treatment of type 2 diabetes

Type 2 diabetes is characterized both by insulin resistance and decreased insulin production, i.a. due to increased β-cell apoptosis. Sustained mTORC1 activity leading to ER stress and JNK activation caused insulin resistance, while treatment with chemical chaperones prevented the ER stress response and enhanced insulin signaling in TSC-deficient cells [39]. Moreover, reducing ER stress in ob/ob mice using these chaperones *in vivo* normalized blood glucose and enhanced insulin sensitivity [45]. Several studies suggested that nutrient overload leads to mTORC1 activation, thereby increasing insulin resistance [46], [47]. Thus, there seems to emerge a consensus that nutrient overload causes insulin resistance by stimulating mTORC1, leading to ER stress and increased JNK activity. Similar mechanisms may also be operative in β-cells. It was previously shown that activation of mTORC1 in β-cells by high glucose or growth factors increased IRS2 degradation with β-cell apoptosis as a consequence [37]. Thus, it is possible that mTORC1 impairs β-cell survival in type 2 diabetes both by increasing IRS2 degradation and by enhancing the stress response to glucose and fatty acids. It remains to be shown whether impaired IRS2 or IRS1 signaling in response to hyperglycemia and possibly to FFAs is mediated through induction of ER stress in β-cells.

Disruption of TSC2 in β-cells increased β-cell size and proliferation, resulting in β-cell mass expansion and improved glucose tolerance [48]. Rapamycin abolished these effects indicating that TSC2 regulates β-cell mass in an mTORC1-dependent manner. Moreover, we have shown that activation of mTORC1 by glucose is important for β-cell adaptation to hyperglycemia in type 2 diabetes: rapamycin treatment of diabetic *P. obesus* markedly increased their hyperglycemia and β-cell apoptosis [38]. This may suggest that mTORC1 has a dual effect in the regulation of β-cell mass in type 2 diabetes: on one hand it is required for the adaptive increase in β-cell size and proliferation by stimulating protein synthesis; and on the other, it may increase ER stress and apoptosis in β-cells, e.g. in response to fatty acids.

Consistent with the contention that mTORC1 has a dual role in the regulation of β-cell mass, mice lacking TSC2 in islets exhibit a biphasic response of their β-cell mass [49]. During early life the β-cell mass of these mice was increased; however, at older age they developed progressive hyperglycemia and hypoinsulinemia accompanied by a reduction in β-cell mass. Thus, different parameters, including the duration of mTORC1 activation, the age of the animals and their metabolic state may influence the β-cell response to modulation of mTORC1 activity. mTORC1 was shown to regulate mitochondrial biogenesis and function [50] in addition to its regulation of ER function; thus, inhibition of mTORC1 may have various effects on different pathways involved in cellular stress.

In summary, mTORC1 probably leads to both insulin resistance and β-cell apoptosis under glucolipotoxic conditions by promoting ER stress. Although inhibition of mTORC1 may alleviate the ER stress response to glucose and fatty acids, prolonged treatment of diabetic animals with mTORC1 inhibitors leads to increased β-cell apoptosis and progressive hyperglycemia [38]. The complex role of mTORC1 in the regulation of β-cell proliferation and apoptosis, as well as in nutrient sensing and metabolism, suggests that inhibition of mTORC1 is unlikely to become a therapeutic approach in type 2 diabetes.

## Materials and Methods

### Islet isolation, β-cell line culture and experimental protocols

Diabetes-prone, 2.5–3.5 month-old male *P. obesus* (Hebrew University Colony, Harlan, Jerusalem, Israel) were fed a low-energy (LE; 2.38 kcal/g) diet (Koffolk, Petach-Tikva, Israel), which maintains normoglycemia (3–5 mmol/l in this species). Islets were isolated from prediabetic, normoglycemic *P. obesus* by collagenase digestion (Collagenase P; Roche Diagnostics GmbH, Mannheim, Germany) as described [51]. The islets were used after repeated washes with Hanks' balanced salt solution. They were cultured in suspension in RPMI 1640 medium (Biological Industries, Beit-Haemek, Israel) with 10% fetal bovine serum, 100 U/ml penicillin, 100 µg/ml streptomycin, 2 mmol/l L-glutamine (Biological Industries) and different glucose concentrations. Animal use was approved by the Institutional Animal Care and Use Committee of the Hebrew University and the Hadassah Medical Organization.

INS-1E β-cells were grown in RPMI 1640 medium (Biological Industries, Beit Haemek, Israel) supplemented with 10% fetal bovine serum, 1 mmol/l sodium pyruvate, 2 mmol/l L-glutamine, 10 mmol/l HEPES, 0.05 mmol/l 2-mercaptoethanol, 100 units/ml penicillin and 100 µg/ml streptomycin.

To study the effects of glucose and palmitate on islet and β-cell ER stress, *P. obesus* islets or INS-1E cells were incubated in RPMI medium with 0.5% (w/v) BSA with or without 0.5 mmol/l palmitate at 3.3 and 22.2 mmol/l glucose for different periods of time. The palmitate-BSA solution was prepared as described [4]. Briefly, the sodium salt of palmitic acid was dissolved at a concentration of 10 mmol/l in 11% BSA in a shaking water bath at 37°C for 16 h. The pH of the palmitate-BSA solution was adjusted to 7.4 with 1 N NaOH. It was then filtered through a sterile 0.2 µ filter and stored at −20°C. The palmitate-BSA solution was diluted 1∶20 in the INS-1E incubation medium before use. The molar ratio of palmitate∶BSA was 6∶1. To assess the role of mTORC1 in palmitate-induced ER stress, *P. obesus* islets or INS-1E cells were treated with the mTORC1 inhibitor rapamycin (50 nmol/l) according to the experimental protocol. Cycloheximide (0.2 mmol/l) was used to study the effects of protein biosynthesis inhibition on ER stress. The JNK inhibitor SP600125 (20 nmol/l) was used to study the role of the JNK pathway in β-cell apoptosis in response to high glucose and palmitate.

INS-1E and islet extracts were used for analysis of mTORC1 signaling, protein biosynthesis, ER stress markers and apoptosis, as described below. All reagents were purchased from Sigma (Rehovot, Israel).

### Anisomycin and thapsigargin treatment of TSC2^−/−^ mouse embryonic fibroblasts

TSC2^−/−^ and control wild-type mouse embryonic fibroblasts were kindly provided by Dr. David J. Kwiatkowski (Harvard Medical School, Boston). Cells were cultured in DMEM (Beit Haemek) supplemented with 10% fetal calf serum, 2 mmol/l glutamine, 100 units/ml penicillin and 100 µg/ml streptomycin. The cultures were treated with 200 nmol/l anisomycin (Sigma) for 30 min, or 300 nmol/l thapsigargin (Sigma) for 24 h with and without 50 nmol/l rapamycin. Cells treated with anisomycin and rapamycin were pre-loaded with rapamycin for 2 h prior to the treatment with the different inducers of JNK. MEFs extracts were used for analysis of JNK and c-Jun phosphorylation and additional markers of ER stress.

### Western blot analysis

Protein expression and phosphorylation in INS-1E cells, *P. obesus* islets and MEFs were studied by standard Western blot analyses using antibodies against p70S6 kinase, phospho-p70S6 kinase (Thr389), S6 ribosomal protein, phospho-S6 ribosomal protein (Ser235/236), 4EBP1 and phospho-4EBP1 (Cell Signaling Technology, Beverly, MA). ER stress markers were assessed using antibodies against total and phospho-IRE1α (kindly provided by Dr. Urano, University of Massachusetts, Boston, MA), phospho-SAPK/JNK (Thr183/Tyr185), SAPK/JNK, c-JUN, phospho-eIF2α total and phospho-PERK (all from Cell Signaling Technology), GADD153/CHOP and eIF2α (Santa Cruz, Biotechnology, Santa Cruz, CA). Molecular activation of apoptosis was assessed by measurement of cleaved caspase 3 using polyclonal goat anti-rabbit antibody (Cell Signaling Technology). Peroxidase-conjugated AffiniPure goat anti-rabbit from Jackson Immunoresearch Laboratories (West Grove, PA) was used as secondary antibody. X-ray film densitometry was used for quantification (ImageMaster VDS-CL, Amersham Pharmacia Biotech, UK).

### Quantitative real-time RT-PCR

RNA was extracted from INS-1E cells using Trireagent (Biolab, Jerusalem, Israel); samples of 1 µg total RNA were reverse transcribed using Moloney murine leukemia virus reverse transcriptase (Promega, Madison, WI, USA). Quantitative real time RT-PCR (qPCR) for IRE1α and spliced Xbp1 was performed on a Prism 7000 Sequence Detection System using the Power SYBR Green PCR Master Mix (Applied Biosystem, Foster City, CA, USA). All samples were analysed in triplicate and corrected for GAPDH used as an internal control. The following oligos were used for the PCR reaction: Spliced Xbp1: forward 5′- GAGTCCGCAGCAGGTG -3′, reverse 5′ -GAA GAG GCA ACA GCG TCA GA -3′; Ire1α: forward 5′- TGTCCCACTTTG TGTCCA ATGG -3′ reverse 5′ - TTGCTCTTGGCCTCTGTCTCCTT -3′; GAPDH: forward 5′- AGT TCA ACG GCA CAG TCA AG -3′ reverse 5′ – TAC TCA GCA CCA GCA TCA CC -3′.

### Apoptosis ELISA

Cells were plated in 96-well plates and grown in RPMI 1640 containing 11.1 mmol/l glucose until reaching 70% confluence. The cells were then exposed for 16 h to 0.5% (w/v) BSA with or without 0.5 mmol/l palmitate at 3.3 and 22.2 mmol/l glucose and different treatments as indicated in the figure legends. Cells were lysed and oligonucleosomes in the cytosol indicative of apoptosis-induced DNA degradation were quantified using the Cell Death ELISA^PLUS^ assay (Roche Diagnostics, Manheim Germany) according to the manufacturer's instructions.

### β-cell proliferation

β-Cell proliferation was analyzed by measuring ^3^H-thymidine incorporation in INS-1E cells. Cells were seeded in 24-well plates and grown to 50–60% confluence. Medium was then changed to RPMI 1640 containing 11.1 mmol/l glucose and 1% BSA. After 24 h, the cells were treated with and without palmitate at 3.3 and 22.2 mmol/l glucose with and without rapamycin in the presence of 1% fetal bovine serum, 1 µCi ^3^H-thymidine and 10 nmol/l cold thymidine for 16 h. The cells were then washed with PBS, treated with 10% trichloroacetic acid for 10 min and repeatedly washed with 5% trichloroacetic acid, followed by cell solubilization with 0.5 ml 0.1 N NaOH. The solubilized cell solution was neutralized with HCl, collected into scintillation vials and radioactive counts measured in a β-counter. Similarly treated cells without radioactive labeled thymidine, were used for cell counting.

### Proinsulin and total protein biosynthesis

For measurement of proinsulin and total protein biosynthesis in INS-1E cells, 5×10^5^ cells were seeded in 12-well plates and treated with 0.5% BSA with and without palmitate at 3.3 and 22.2 mmol/l glucose with and without rapamycin or cycloheximide for 16 h. The last 2 h of the incubations were performed in KRBH-BSA buffer containing similar treatments and 10 µCi L-[2, 3, 4, 5-^3^H]leucine (120 Ci/mmol; ARC, St. Louis, MO). After 2-h incubation at 37°C, leucine incorporation was terminated by ice-cold wash-out in glucose-free KRBH-BSA buffer. The cells were then scrapped and centrifuged and the pellets subjected to immunoprecipitation, as described [52]. Briefly, the INS-1E cell pellets were suspended in 450 µl 0.2 mol/l glycine buffer containing 0.1% RIA-grade BSA and 0.5% NP-40, pH 8.8 (GB/NP40 buffer), and subjected to four freeze-thaw cycles in liquid nitrogen. Each sample (50 µl) was pretreated with protein A-Sepharose (Sigma) before immunoprecipitation with anti-insulin serum (Sigma, product number: I8510), to correct for nonspecific binding. Comparing the results obtained by immunprecipitation to those obtained by HPLC analysis validated the accuracy of the proinsulin biosynthesis analysis (Figure S4). Total protein biosynthesis was determined by trichloroacetic acid precipitation [53].

### Data presentation and statistical analysis

Data shown are means±SE. Statistical significance of differences between groups was determined by one-way ANOVA followed by Newman-Keuls test using the InStat statistical program from GraphPad Software, Inc. (San Diego, CA). A paired-sample *t* test was used when the difference between a reference (taken as 100%) and a test was analyzed. A *P* value of less than 0.05 was considered significant.

## Supporting Information

Figure S1 Effects of glucose, palmitate and rapamycin on β-cell proliferation. INS-1E cells were treated with and without palmitate at 3.3 and 22.2 mmol/l glucose with and without rapamycin in the presence of 1% fetal bovine serum, 1 µCi ^3^H-thymidine and 10 nmol/l cold thymidine for 16 h. Thymidine incorporation was determined as described in the Materials and Methods. Each experiment was performed in triplicates. Results are expressed as means±SE. ^∧^ p<0.001 for the difference between the indicated groups.
(0.10 MB TIF)

Figure S2 Nutrients and rapamycin effects on IRE1α protein and mRNA levels at different time points. INS-1E cells were incubated at 3.3 and 22.2 mmol/l glucose with and without 0.5 mmol/l palmitate and 50 nmol/l rapamycin for 4, 8 and 16 h. (A) IRE1α protein expression was analyzed by Western blot. A representative gel of 3 individual experiments is shown; (B) Quantification of IRE1α mRNA levels under the different treatment conditions at 4 h. Results are expressed as means±SE of 4 individual experiments.
(0.18 MB TIF)

Figure S3 Effect of JNK inhibition on glucose and palmitate-induced ER stress. INS-1E cells were incubated for 16 h at 3.3 and 22.2 mmol/l glucose with 0.5% BSA with and without 0.5 mmol/l palmitate and 20 nmol/l of the JNK inhibitor SP600125. The effect of JNK inhibition on c-Jun, JNK and PERK hosphorylation, and on CHOP expression was analyzed by Western blot; a representative gel is presented (A). Quantification of PERK phosphorylation and CHOP expression is shown in (B). Results are expressed as means±SE of 4 individual experiments. * p<0.05, ^∧^ p<0.001 for the difference between the indicated groups.
(0.45 MB TIF)

Figure S4 Comparison of proinsulin biosynthesis measurements by immunoprecipitation and HPLC. Rat islets were treated with 16.7 mmol/l glucose (G16.7) or 10 mmol/l succinate (SAM) for 1 h followed by metabolic labeling with L-[2, 3, 4, 5-^3^H]leucine. Islet extracts were subjected to either immunoprecipitation using anti-insulin serum as described in the Material and Methods or HPLC analysis (for details see Gadot et al, Endocrinology 136:4218–4223, 1995). A representative HPLC profile is shown above and a comparison between the proinsulin measurements obtained with the two assays is shown below.
(0.45 MB TIF)
