# Supplementary figures and images for: Glucose Amplifies Fatty Acid-Induced Endoplasmic Reticulum Stress in Pancreatic β-Cells via Activation of mTORC1
Source: PLoS One. 2009 Mar 23;4(3):e4954. doi: 10.1371/journal.pone.0004954 (PMC2654723; doi:10.1371/journal.pone.0004954)

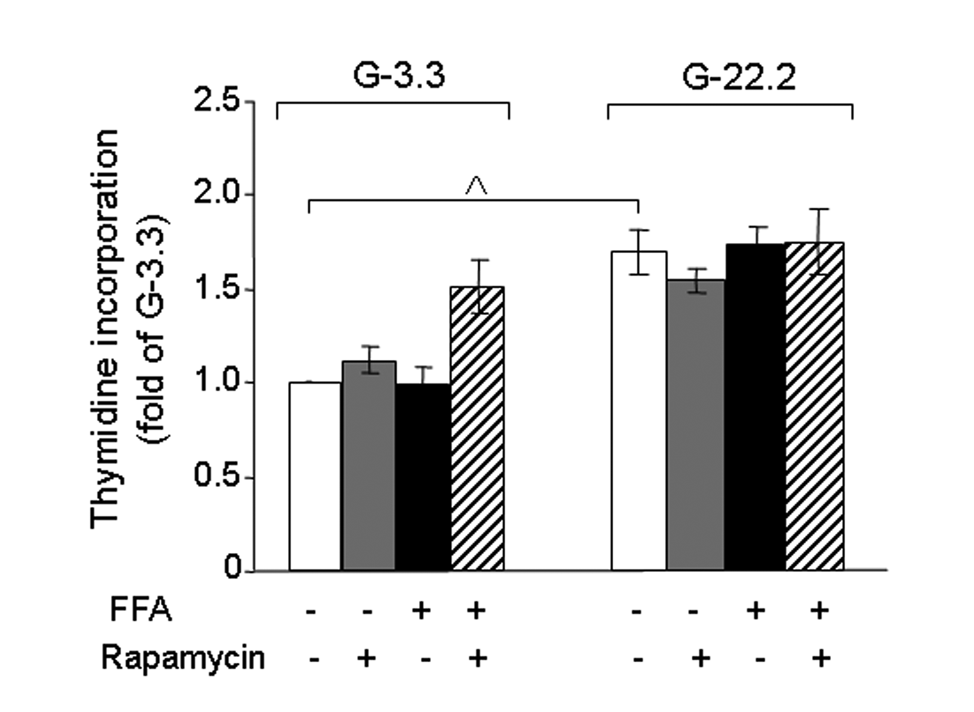

Supplement: Figure S1 — Effects of glucose, palmitate and rapamycin on β-cell proliferation. INS-1E cells were treated with and without palmitate at 3.3 and 22.2 mmol/l glucose with and without rapamycin in the presence of 1% fetal bovine serum, 1 µCi 3H-thymidine and 10 nmol/l cold thymidine for 16 h. Thymidine incorporation was determined as described in the Materials and Methods. Each experiment was performed in triplicates. Results are expressed as means±SE. ∧ p<0.001 for the difference between the indicated groups. (0.10 MB TIF) [file pone.0004954.s001.tif]

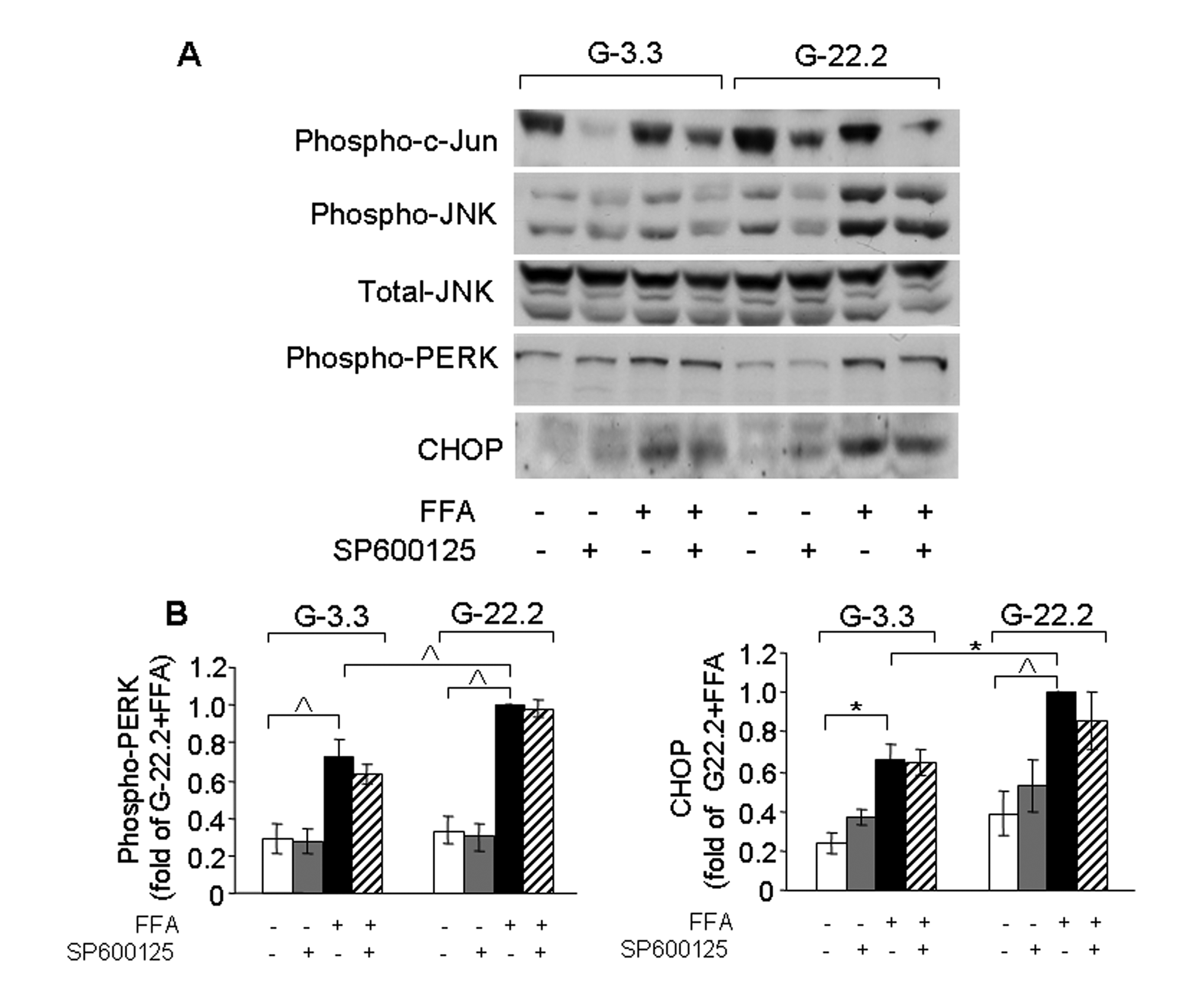

Supplement: Figure S3 — Effect of JNK inhibition on glucose and palmitate-induced ER stress. INS-1E cells were incubated for 16 h at 3.3 and 22.2 mmol/l glucose with 0.5% BSA with and without 0.5 mmol/l palmitate and 20 nmol/l of the JNK inhibitor SP600125. The effect of JNK inhibition on c-Jun, JNK and PERK hosphorylation, and on CHOP expression was analyzed by Western blot; a representative gel is presented (A). Quantification of PERK phosphorylation and CHOP expression is shown in (B). Results are expressed as means±SE of 4 individual experiments. * p<0.05, ∧ p<0.001 for the difference between the indicated groups. (0.45 MB TIF) [file pone.0004954.s003.tif]

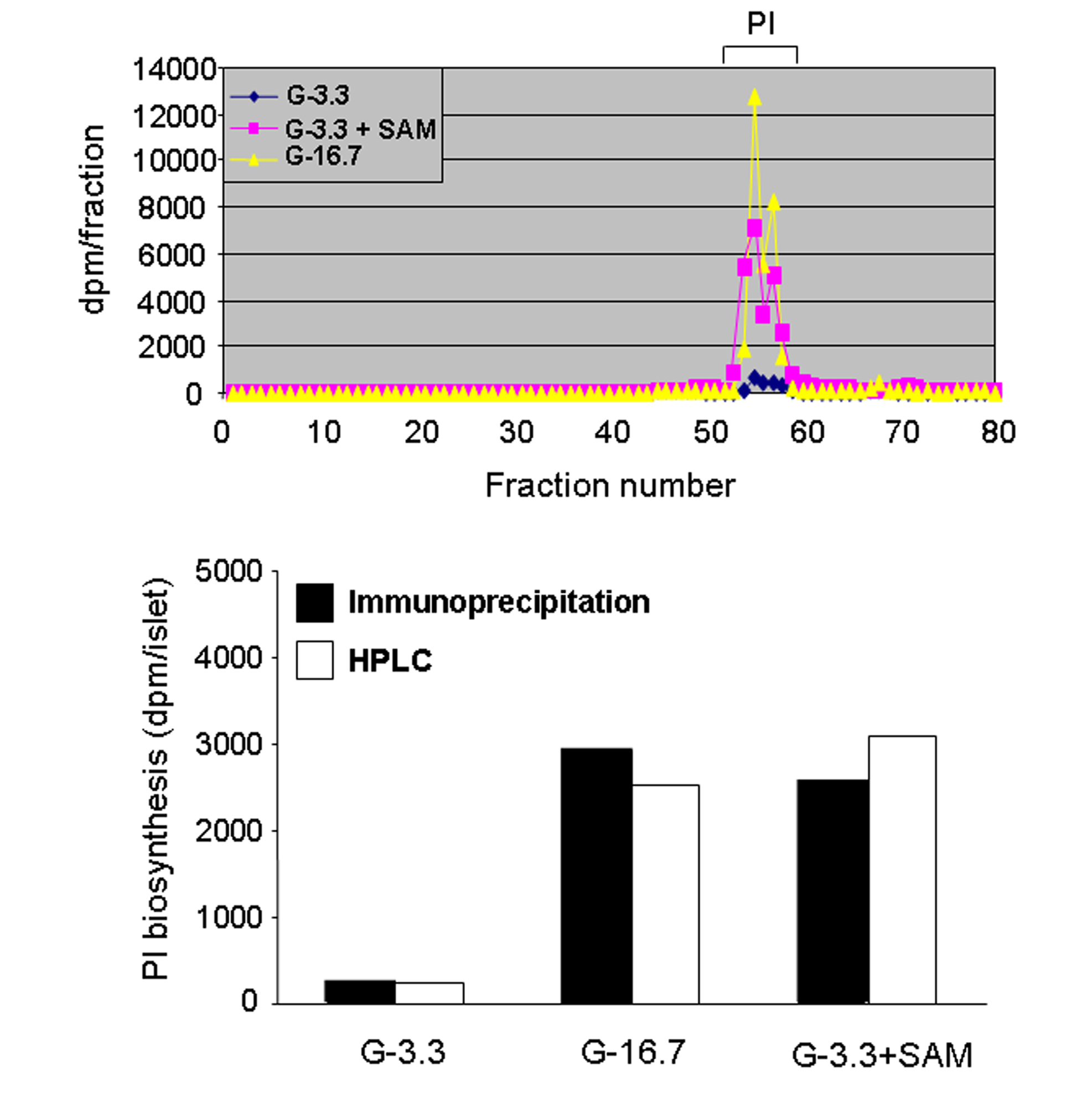

Supplement: Figure S4 — Comparison of proinsulin biosynthesis measurements by immunoprecipitation and HPLC. Rat islets were treated with 16.7 mmol/l glucose (G16.7) or 10 mmol/l succinate (SAM) for 1 h followed by metabolic labeling with L-[2, 3, 4, 5-3H]leucine. Islet extracts were subjected to either immunoprecipitation using anti-insulin serum as described in the Material and Methods or HPLC analysis (for details see Gadot et al, Endocrinology 136:4218–4223, 1995). A representative HPLC profile is shown above and a comparison between the proinsulin measurements obtained with the two assays is shown below. (0.45 MB TIF) [file pone.0004954.s004.tif]
